# Physiological Metrics of Surgical Difficulty and Multi-Task Requirement during Robotic Surgery Skills

**DOI:** 10.3390/s23094354

**Published:** 2023-04-28

**Authors:** Chiho Lim, Juan Antonio Barragan, Jason Michael Farrow, Juan P. Wachs, Chandru P. Sundaram, Denny Yu

**Affiliations:** 1 School of Industrial Engineering, Purdue University, West Lafayette, IN 47907, USA; lim302@purdue.edu (C.L.);; 2 School of Medicine, Indiana University, Indianapolis, IN 46202, USA

**Keywords:** cognitive workload, multitask, multimodality, physiological signal, surgical skill

## Abstract

Previous studies in robotic-assisted surgery (RAS) have studied cognitive workload by modulating surgical task difficulty, and many of these studies have relied on self-reported workload measurements. However, contributors to and their effects on cognitive workload are complex and may not be sufficiently summarized by changes in task difficulty alone. This study aims to understand how multi-task requirement contributes to the prediction of cognitive load in RAS under different task difficulties. Multimodal physiological signals (EEG, eye-tracking, HRV) were collected as university students performed simulated RAS tasks consisting of two types of surgical task difficulty under three different multi-task requirement levels. EEG spectral analysis was sensitive enough to distinguish the degree of cognitive workload under both surgical conditions (surgical task difficulty/multi-task requirement). In addition, eye-tracking measurements showed differences under both conditions, but significant differences of HRV were observed in only multi-task requirement conditions. Multimodal-based neural network models have achieved up to 79% accuracy for both surgical conditions.

## 1. Introduction

Minimally invasive surgery (MIS) has several important benefits; these include smaller incisions, better perioperative pain control, reduced scar burden, and less blood loss [1,2,3]. While the purported benefits to the patient are well-established, the physical challenges encountered by the surgeon are less well defined. MIS approaches utilizing conventional laparoscopy have induced physical pain, fatigue, and high cognitive workload for surgeons secondary to lack of depth perception and restricted movement of laparoscopic tools [4,5,6,7]. Robotic-assisted surgery (RAS) was developed to ameliorate the aforementioned challenges by providing an ergonomic interface while maintaining the patient-level benefits of laparoscopy. In contrast to conventional laparoscopy, during RAS, surgeons perform key elements of the procedure remotely within a console equipped with two master controllers. The visual magnification to support the added dexterity afforded by the robotic platform allows for complex procedures to be safely completed while overcoming some of the known limitations of conventional laparoscopy [4]. However, technical advances can result in unintended consequences to both the patient and the surgeon. Specifically, the added cognitive workload experienced by surgeons during RAS due to remote positioning from the patient, necessitating optimal communication with the bedside assistant, the coordinated use of multiple instruments simultaneously on a limited visual field, and the lack of any tactile feedback can generate significant challenges that may ultimately affect patient safety.

This added workload can become excessive when the various tasks exceed an individual’s perceived resources to cope [8,9]. Information overload has also been correlated with poorer performance due to limitations on human information processing ability [10]. These findings have been demonstrated in similarly high-stakes professions such as aviation and the military [11,12]. Surgeons continuously face demanding tasks that are inherently multifactorial and are necessarily time-sensitive. The abundance of situational data input could produce deleterious cognitive workloads that may result in performance errors [13,14]. 

Prior efforts at understanding this phenomenon were built upon self-reported measurements (Multiple Resources Questionnaire/NASA Task Load Index), which demonstrated a decrease in cognitive workload during RAS when compared to conventional laparoscopy [15,16,17,18,19]. However, these self-reported workload measurements are limited by significant biases and may be less reliable than objective measures [20,21]. To tackle these limitations, sensor-based measurements are being explored, and most recently, some studies have employed electroencephalography (EEG) sensors to assess cognitive states [22,23]. For example, better RAS performance in lysis of adhesions surgery was achieved when surgeons utilized more mental resources measured by EEG [23]. Multimodal-sensing approaches have also been proposed for the detection of different cognitive states between pre-and post-RAS training: EEG and eye-tracking [24] and EEG, heart rate variability (HRV), electromyography, and electrodermal activity [25]. Notably, multimodal sensing outperformed individual sensors for predicting cognitive load.

These previous RAS-related studies have focused on the surgical task difficulty to control participants’ cognitive states [15,16,17,18,19,24,25]. Specifically, as primary task difficulty increases, so do the processing resources required, ultimately exceeding the capacity available and resulting in deteriorating performance [26]. However, many errors, in particular skill-based errors (i.e., ‘slips’ and ‘lapses’), occur when there are distractions and interruptions. In addition, the cognitive workload can vary depending on the number of concurrent tasks to be processed [27,28]. In the operating room, surgeons are continuously and inevitably facing situations where multiple clinical inputs and outputs occur concurrently. These divided attention tasks in dual-task interference (or multi-task requirement) are difficult to isolate and understand based on previous studies that only defined cognitive load by task difficulty. Thus, the cognitive workload caused by processing simultaneous surgical tasks needs to be studied to examine how the multi-task requirements influence physiological responses during RAS under different levels of surgical task difficulties.

To better understand how physiological responses represent the cognitive workload encountered in various RAS conditions, we aimed to examine the synchronous effects of two factors: primary task (i.e., surgical task difficulty) and multi-task requirement. We hypothesized that physiological measurements could discriminate the degree of cognitive workloads from both study factors, but that the physiological responses induced from each study factor are different. Our secondary objective was to determine the accuracy of machine learning models for predicting the study factors using the physiological features that can be extracted from multimodal physiological sensors.

## 2. Materials and Methods

This research has been reviewed and approved by University’s Institutional Review Board (IRB-1906022354).

### 2.1. Participants

A total of 10 engineering university students (8 males/2 females) with no prior experience in surgical-related tasks were recruited for our multi-session repeated measures study. Two participants’ heart rate variability (HRV) data were removed due to disconnection of the HRV sensor during the four sessions. Thus, 10 participants’ data were used to analyze EEG and eye-tracking, and 8 participants’ data were used for the HRV analysis and development of classification models. All participants were right-hand dominant with a mean age of 25 ± 1.2 years. The participants provided written consent and were paid for their participation.

### 2.2. Experimental Design

To elicit different cognitive states in the users, we designed an experiment with two factors: primary task and multi-task requirement. In the primary task factor, we examined the effects of tasks requiring different degrees of cognitive processing resources; the multi-task requirement factor allowed us to study the effects of distractors and spare working memory as participants performed secondary tasks alongside primary tasks during simulated RAS. We adopted the subsidiary task paradigm for the multi-task requirement where participants were required to maintain primary task performance at the expense of the secondary task [26]. Participants carried out four-session experiments over two days. Each session consisted of a combination of two primary tasks (peg transfer/suturing) and multi-task requirement conditions (single task condition/n-back/target game) in random order. Participants performed six tasks in each session (Table 1). Thus, the participants were required to have four repeated measures for each of the six tasks, resulting in a total of 24 tasks for each participant.

#### 2.2.1. Primary Task

The primary task contained two levels. Each level was a different task with different workload requirements. Peg transfer and suturing tasks were adapted from the Fundamentals of Laparoscopic Surgery (FLS) training program, developed by the Society of American Gastrointestinal and Endoscopic Surgeons and launched in 2004 [29]. These tasks had different levels of hand-eye coordination and bimanual dexterity as described below:

- The peg transfer task required the participants to translocate six objects to the peg. Participants started to grasp the object with the non-dominant hand from the left side of the pegboard and transferred it to the dominant hand to place on the right side of the board (if the left hand is a dominant hand, the task starts from the right side of pegboard). Once six objects were entirely transferred, the objects on the right side were transferred to the left side of the board, starting with the dominant hand. The participants were required to repeat the peg transfer task for up to 3 min.
- For the suturing task, a cloth material pad had a slit to guide the suturing path. The suturing task required the participants to put the needle precisely in one side of the slit and put it through on the other side of the slit using the robot’s gripper, which required them to constantly adapt the wrist position of the end effector. Along with this, the interval of stitching needed to be consistent. The participants were required to repeat the suturing task for up to 3 min.

#### 2.2.2. Multi-Task Requirement 

To assess spare working memory capacity, users were instructed to perform secondary tasks. Secondary tasks are based on the Multiple Resource Theory (MRT) [30,31] and are widely used to assess cognitive workload. From the MRT, a secondary task uses the remaining mental capacity after a primary task takes a certain amount of mental capacity. In most cases, the successful performance relies on how well the operator retrieves information from memory. In particular, the working memory has been closely related to the operator’s performance in a variety of situations [26]. In this study, auditory and visual working memory tasks that are frequently engaged with primary surgical procedures were adopted to measure sensory-related multi-task effects on physiological responses. As the user performed the primary task, three multi-task requirement conditions were studied: single task condition (only peg transfer or suturing), n-back multi-task, or a target game multi-task coded in the da Vinci Research Kit (dVRK, Intuitive Surgical Inc., Sunnyvale, CA, USA). 

The n-back task has been used to investigate working memory in dual-task experiments [32]. This study adopted the auditory 2-back task [33] as an auditory memory task instead of the traditional visual n-back. The visual n-back can block or interfere with the view of the main surgical task. Pre-recorded sets of single-digit numbers (0–9) were randomly presented verbally at 26.6 wpm. Following established n-back protocol, participants recited numbers heard two positions previously throughout the task.

**Figure 1:**
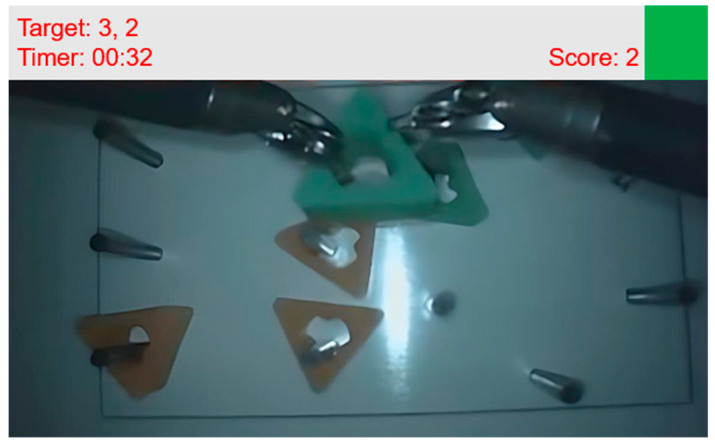
An example of a stereo viewer which has target game information during a peg transfer task. There are two target numbers (3 and 2). The participants were instructed to tap the foot pedal each time the timer’s second digit number corresponded to the target numbers. (In the example, the timer displays 00:32. The second digit is 2.) Once the participants play the game successfully, a green square in the upper corner of the stereo display appears and the score is increased by 1.

The target game as a visual memory task was designed by adjusting the Sternberg memory search task (item-recognition task) [34] to dVRK. A timer (mm:ss) with one-digit target numbers was displayed in the dVRK stereo viewer. Figure 1 shows the example of a stereo viewer including target game information. The users were instructed to tap the console foot pedal each time the timer’s second digit number corresponded to the target numbers. The random target numbers changed every 20 s. The target game information was displayed at the top of the stereo display to avoid blocking the view of the main task. Based on the Sternberg memory search task [34], the target number, second digit number of the timer, and pedal tapping were considered positive stimulus set, test stimulus, and positive response, respectively.

### 2.3. Data Analysis

#### 2.3.1. Electroencephalogram (EEG)

A wireless electroencephalogram (EEG), g.Nautilus (Guger Technologies OG, Graz, Austria), was employed to collect EEG signals. Signals were sampled at 250 Hz on 32 channels positioned based on a 10–20 system. This device used an ear-clip electrode as a reference. The collected EEG data were preprocessed using EEGlab in MATLAB [35]. The EEG data were filtered offline using a basic finite impulse response filter of 0.1–30 Hz and the data were re-referenced to the average of the signal [36]. Cleanline plugin was used for removing sinusoidal noise, specifically, power line interference in EEG recordings [37]. To remove artifacts generated by eye movements, eye blinks, and temporal muscle activities, independent component analysis (ICA) [35] and semi-automated rejection plugin (ADJUST) were used [38]. 

After artifact removal, EEG signals were processed using spectral analysis to quantify band power, and EEG indices were calculated to assess cognitive workload [39,40,41,42]. Power spectral density analysis was computed using Welch’s method with a Hanning window of 0.2 s and 50% overlap for the 3-min task duration data of each channel to calculate absolute band power (theta (4–8 Hz)/alpha (8–12 Hz)/beta (12–30 Hz)). The formulas for calculating the engagement index and theta-alpha ratio (TAR) were calculated using Equations (1) and (2), respectively. In this study, we used EEG data from P3, P4, PZ, and CZ channels to calculate the engagement index as proposed by Prinzel et al. [43]. The theta band power of the FZ channel and alpha band power of the PZ channel were used to calculate TAR as proposed by Holm et al. [44].

Engagement Index = (beta band power)/(theta band power + alpha band power)(1)

TAR = (theta band power (FZ))/(alpha band power (PZ))(2)

#### 2.3.2. Eye-Tracking

Wearable eye-tracker, Tobii Pro Glasses 2 (Tobii Technology AB, Danderyd, Sweden), was used to monitor participants’ gaze data during the experiments. Data were collected at 50 Hz. The Tobii Pro Lab Software (Tobii Technology AB, Danderyd, Sweden) was used to extract the fixation and saccade metrics. In addition, Saccade-Fixation Ratio (SF Ratio) [45] and gaze entropy were extracted from the eye-tracking data. SF ratio was calculated using Equation (3) [46], and gaze entropy was calculated following Equation (4) [47]:

(3) SF ratio = (Total Saccade Time)/(Total Fixations Time)

(4) $$H\left(X\right)\;=\;-\sum p\left(x,\;y\right)\log_{2}p(x,\;y)$$

where p(x, y) is the probability of the gaze falling in the (x, y).

#### 2.3.3. Heart Rate Variability (HRV)

This study used optical pulse ear clip (PPG) of the Shimmer3 GSR + Unit (Shimmer, Dublin, Ireland) to acquire HRV data. The data were collected at 128 Hz. Time and frequency domain metrics were extracted from the HRV data and used as indicators of workload. The Kubios HRV Premium software (Kubios Oy, Kuopio, Finland) was used to extract the time domain metrics (mean RR interval (meanRR), standard deviation of RR (stdRR), the square root of the mean squared differences between adjacent normal RR interval (RMSSD), the percentage of adjacent NN intervals that differ from each other by more than 50 ms (pNN50)) and frequency domain metrics (low frequency (LF, 0.04–0.15 Hz)/high frequency (HF, 0.15–0.4 Hz) ratio). 

### 2.4. Procedure

Prior to the experiments, each participant was trained on the dVRK to become familiar with the primary tasks (peg transfer/suturing) and the multi-task requirement tasks (n-back/target game) for 3 h over two days. After the training sessions, we conducted a basic suturing task within a 3-min limit to screen the participants who did not have a sufficient skill level. Three participants were excluded from this task. In this study, we utilized the first generation Da Vinci robot with the proprietary software and hardware provided by the dVRK development community [48]. After the training session, participants performed the four study sessions as outlined in the experimental design. Physiological data were collected throughout the sessions with the aforementioned EEG, eye-tracker, and HRV sensors. Each sensor was set up and calibrated based on the manufacturer’s guidelines.

### 2.5. Statistical Analysis

Statistical analyses were performed in R Studio (version 1.3.959, R-core 4.0.1). Prior to the analyses, the normality test of the data was conducted, and outliers were removed from datasets (EEG/eye-tracking/HRV) using Cook’s distance measure [49]. The extracted features were examined using a linear mixed model [50], with the primary task (peg transfer/ suturing) and multi-task requirement (single task condition/n-back/target game) as fixed variables and subjects as random effects. Two-way interactions were explored, and Tukey’s multiple comparison test was further performed to determine differences between factor level combinations at the significance level of 0.05.

### 2.6. Artificial Neural Network Classification Model

Classification models were developed for a supervised binary class problem. The classification model consists of two hidden layers and one output layer. A log-sigmoid transfer function was used in hidden layers, and a softmax function was used for an output layer [51].

The extracted features from physiological sensors were used as input to develop the artificial neural network. To reduce redundancy and improve the accuracy and generalization capability of the classification model, the least absolute shrinkage and selection operator (Lasso) was used to identify the key feature subset of input variables [52]. 

In this study, four classification models were developed based on the study factors: (1) primary task classification model (peg transfer vs. suturing), (2) multi-task requirement classification models (single task condition vs. n-back multi-task, single task condition vs. target game multi-task, n-back multi-task vs. target game multi-task). For evaluation of model performance, 10-fold cross validation was implemented. 

## 3. Results

Physiological data were collected from a total of 10 participants, and each participant completed four sessions (60 min/session) over two days and performed 24 tasks in total. One and three data samples were removed based on the Cook’s distance measure for the EEG and eye-tracking datasets, respectively. The results below are organized by physiological modality and describe how study factors (primary task and multi-task requirement) affected the observed physiological responses. 

### 3.1. Electroencephalogram (EEG)

#### 3.1.1. Effect of the Primary Task

**Figure 2:**
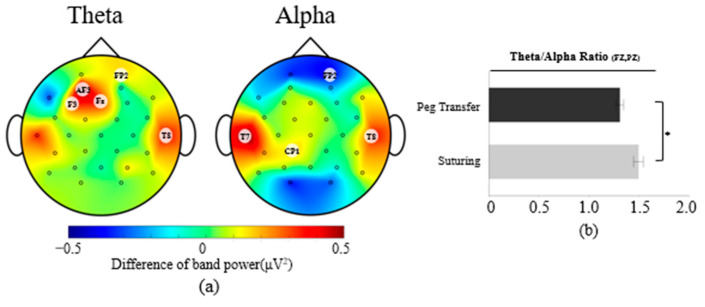
(**a**) EEG topographic plots in accordance with each band power. Red and blue color areas indicate higher and lower band power of the suturing task compared to the peg transfer task, respectively; (**b**) difference of TAR for the effect of the primary task (error bars show standard errors). Asterisk indicates a statistically significant difference with a significance level of 0.05.

Figure 2a shows the EEG topographic plots of the averages of the band power differences between the suturing and peg transfer tasks across all participants, with the band power of peg transfer subtracted from the band power of suturing. Theta band power was found to be significantly higher on five channels during suturing tasks compared to peg transfer tasks, F(1, 239) = [3.88–14.20], *p* < 0.05. A post hoc Tukey test showed that theta band power during the suturing tasks was [0.31–0.59 μV^2^] higher than theta band power during the peg transfer tasks. 

The suturing tasks showed significant increases and decreases of alpha band power on 4 channels (F(1, 239) = [4.03–8.35], *p* < 0.05) compared to peg transfer tasks. From the Tukey test, alpha power during the suturing tasks was 0.16, 0.56, and 0.36 μV^2^ higher than during the peg transfer task on the CP1, T7, and T8 channels, respectively, but 0.38 μV^2^ lower on the FP2 channel. 

For EEG index measures, significantly higher TAR were observed in the suturing tasks, with F(1, 239) = 15.57, *p* < 0.05. From the Tukey test, 0.18 higher TAR was observed during the suturing tasks than the peg transfer tasks (Figure 2b). 

No significant differences were observed for the beta band power and the engagement index between primary tasks. 

#### 3.1.2. Effect of the Multi-Task Requirement

**Figure 3:**
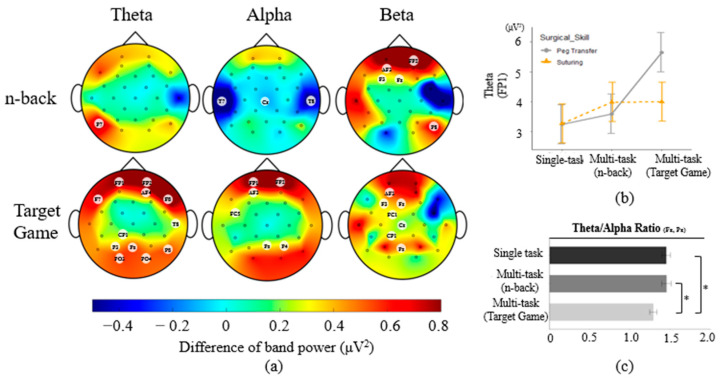
(**a**) EEG topographic plots in accordance with each band power and multi-task requirement (n-back/target game). Red and blue color areas indicate higher and lower band power of multi-task requirement tasks compared to single task conditions, respectively; (**b**) interaction plot of theta (FP1) indicating significant differences between peg transfer with the target game and other levels of peg transfer without the target game and suturing task (error bars show standard errors); (**c**) difference of TAR for the effect of the multi-task requirement (error bars show standard errors). Asterisk indicates a statistically significant difference with a significance level of 0.05.

Figure 3a shows the EEG topographic plots of the averages of the band power differences between multi-task requirement and single task conditions across all participants, with the band power of the single task conditions subtracted from the band power of the multi-task requirement conditions. For the effect of the multi-task requirement, higher theta band power values were observed on 13 channels in the multi-task requirement than in the single task condition (F(2, 239) = [3.19–20.54], *p* < 0.05). Specifically, theta band power on P7 was 0.37 μV^2^ higher for the n-back multi-task than for a single task condition. Theta band power during target game multi-task was [0.31–1.92 μV^2^] higher on 12 channels than theta band power during a single task condition. The multi-task requirement factor had significant interactions with the primary task on the FP1 channel, F(2, 239) = 3.30, *p* < 0.05. The theta power on FP1 was 2.05–2.41 μV^2^ higher when participants performed the peg transfer task simultaneously with the target game than when performing the peg transfer task without the target game. The theta band power of the peg transfer with the target game was 2.30 μV^2^ higher than the theta band power during the suturing task only (Figure 3b). 

The multi-task requirement both increased and decreased alpha band power on 9 channels compared to the single task condition (F(2, 239) = [3.09–10.63], *p* < 0.05). The Tukey test showed that when n-back was added to the primary task, [0.17–1.00 μV^2^] lower alpha band power was observed compared to alpha band power during a single task condition on 3 channels. With the target game, [0.18–0.86 μV^2^] higher alpha power was observed than during a single task condition on 6 channels. 

For beta band power, multi-task requirement (F(2, 239) = [3.21–9.98], *p* < 0.05) had [0.28–3.67 μV^2^] and [0.19–0.57 μV^2^] higher beta band power for n-back (5 channels) and target game multi-task (7 channels), respectively, when compared to the single task condition. 

Among EEG index measures, only TAR differed between task conditions, with F(2, 239) = 5.13, *p* < 0.05. From the Tukey test, TAR was 0.16 lower during the target game multi-task than the single task condition. In addition, the n-back multi-task had 0.16 higher TAR than the target game multi-task. No significant differences were observed for the engagement index.

### 3.2. Eye-Tracking

#### 3.2.1. Effect of the Primary Task

**Figure 4:**
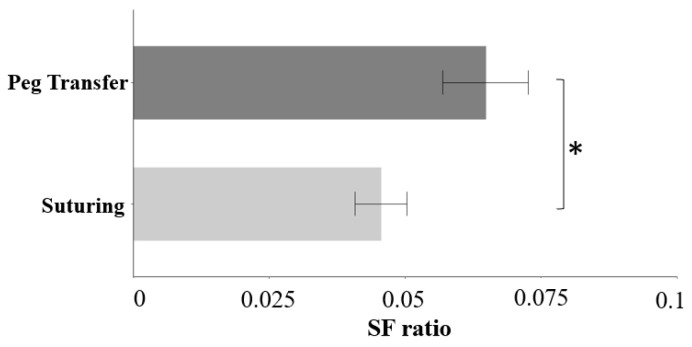
Bar plot of SF ratio for the effect of primary task (error bars show standard errors). Asterisk indicates a statistically significant difference with a significance level of 0.05.

Participants had 0.02 lower SF ratio during the suturing tasks than peg transfer tasks (F(2, 237) = 7.84, *p* < 0.05) (Figure 4). Gaze entropy did not differ between primary tasks. 

#### 3.2.2. Effect of the Multi-Task Requirement

**Figure 5:**
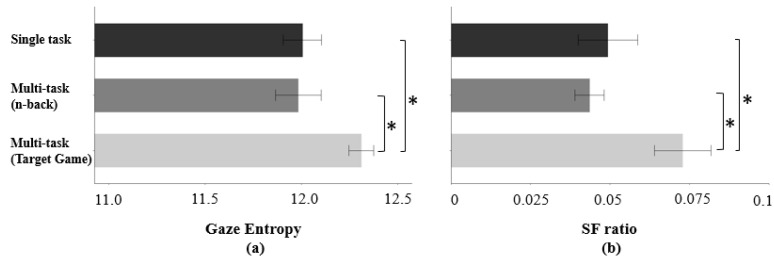
(**a**) Difference of gaze entropy and (**b**) SF ratio across multi-task requirements (error bars show standard errors). Asterisk indicates a statistically significant difference with a significance level of 0.05.

Gaze entropy (F(2, 237) = 4.90, *p* < 0.05) and SF ratio (F(2, 237) = 5.92, *p* < 0.05) differed statistically among three conditions (single task condition/n-back/target game) (Figure 5). A post hoc Tukey test showed that the gaze entropy was 0.30 higher, and the SF ratio was 0.02 higher during the target game multi-task than during the single task condition (Figure 5). Gaze entropy and SF ratio during the target game were 0.33 and 0.02 higher than during the n-back multi-task, respectively (Figure 5). Eye-tracking indicators did not differ between the single task condition and n-back multi-task. 

### 3.3. Heart Rate Variability (HRV)

#### 3.3.1. Effect of the Primary Task

**Figure 6:**
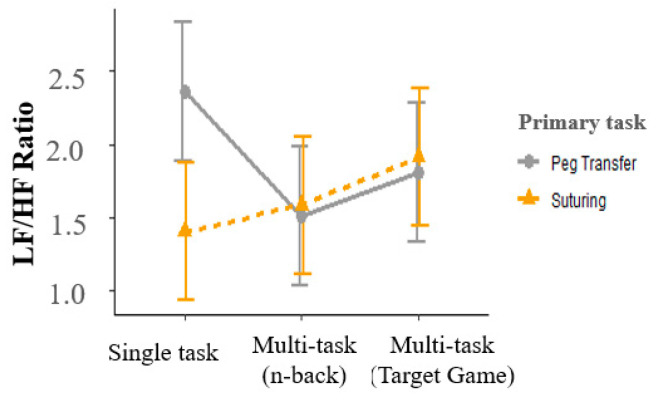
Interaction plot of LF/HF ratio indicating a significant difference between peg transfer and suturing tasks for a single task condition. Error bars show standard errors.

Interaction between primary and multi-tasks was significant for HRV (F(2, 192) = 3.76, *p* < 0.05). Suturing tasks had a 0.96 lower LF/HF ratio than peg transfer tasks for the single-task conditions but did not differ for the multi-task conditions (Figure 6). Other HRV indicators were not sensitive to differences between primary tasks.

#### 3.3.2. Effect of the Multi-Task Requirement

**Figure 7:**
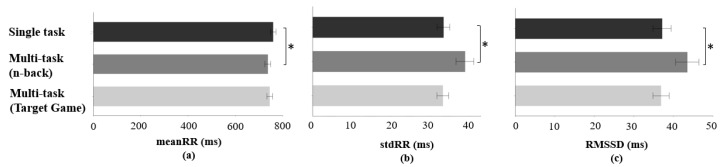
(**a**) Difference of meanRR for effect of multi-task requirement (n-back); (**b**) difference of stdRR for effect of multi-task requirement (n-back); (**c**) difference of RMSSD for effect of multi-task requirement (n-back). Error bars show standard errors. Asterisk indicates a statistically significant difference with a significance level of 0.05.

Significant differences during n-back multi-task in meanRR (F(2, 192) = 4.15, *p* < 0.05), stdRR (F(2, 192) = 6.56, *p* < 0.05), and RMSSD (F(2, 192) = 4.96, *p* < 0.05) were observed (Figure 7) compared to the single task condition. During the n-back multi-task, meanRR was 22.44 ms lower than meanRR during the single task condition. In addition, 5.45 ms higher stdRR and 6.36 ms higher RMSSD were observed during n-back multi-task than during a single task condition. Other HRV indicators were not statistically significant in terms of a multi-task requirement effect.

### 3.4. Artificial Neural Network Classification Model

Eight participants’ data were used to develop classification models, and if one of the physiological data in the specific task was removed based on the Cook’s distance measure, other modality data in the task were not used in the development of the classification model. Accuracy of classification models in distinguishing differences between study factors (primary task effect and multi-task requirement effect) using physiological metrics ranged from 65 to 79%.

The Lasso feature selection algorithm identified the best subset of input variables from three physiological sensors for each classification model (Table 2). The model performances measured by accuracy and F1-score indicated that approximately 65% accuracy was achieved for the primary task effect, 79% for the single task condition vs. n-back multi-task model, 76% for the single task condition vs. target game multi-task model, and 72% for the n-back multi-task vs. target game multi-task model (Table 2).

## 4. Discussion

Cognitive workload varies dynamically and can come from a wide variety of work demands during RAS. Physiological measurements sensitive to these demands are needed to identify the influence of various cognitive workloads encountered during RAS. However, existing studies predicting cognitive workload during RAS have primarily focused on modulating cognitive load using task difficulty. This limits (1) the potential applicability of previously identified behaviors to RAS (where multiple sources contribute to users’ cognitive demands) and (2) our understanding of physiological response behaviors and whether they are sensitive to changes to these demands. In this study, we measured patterns of physiological responses due to changes and interactions in workload demands from the primary task and multi-task requirement study factors. Finally, multimodal physiological signal-based classification models were developed to distinguish the primary tasks and multi-task requirements. The following discussion of the experimental results is organized by physiological modalities.

### 4.1. Electroencephalogram (EEG)

#### 4.1.1. Theta Band Power

Previous work found evidence that theta band power increased with more demanding tasks [53,54]. In our work, the suturing task was designed to require a higher degree of processing resources than peg transfer for the primary task factor. For the multi-task requirement factor, n-back and target game multi-tasks were designed to disperse participants’ attentional resources to a second task and increase the memory load compared to the single task condition.

Theta band power distinguished between the two study factors (primary task and multi-task requirement). Previous studies have shown that the theta band power increases with high cognitive resource demand and high task difficulty [55]. This is consistent with our findings showing higher theta band power during the suturing task than during the peg transfer task. In this study, the suturing task required higher levels of hand-eye coordination and bimanual dexterity than the peg transfer task. For the primary task effect, the increased theta band power was primarily observed over the frontal area during suturing tasks with comparison to theta band power during peg transfer tasks. This result was consistent with the claim that the frontal cortex is linked to cognitive workload for the theta band power [56].

In addition, previous studies found that theta band power increased as the number of concurrent tasks needing to be processed increased [57,58]. For example, Scharinger et al. investigated the effects of the n-back task for theta band power in three conditions (digit value, position, and form). They observed an increase in theta band power as working memory load was increased by the n-back task [59]. This was consistent with our findings with the n-back task. The n-back multi-task increased theta band power over the parietal area. In addition, a significant change in theta band power was observed for the target game multi-task over the frontal and parietal areas. These regional effects can be potentially explained with the existing literature. Working memory has been linked to the parietal area of the brain [60]. For example, in a previous study, with an increased number of visual items (up to 3–4) to memorize [61], the parietal area had more activation. In addition, higher theta band power in the fronto-parietal area reflected lower working memory capacity [62,63,64]. In our study, participants were required to memorize numerical items during the n-back and the target game multi-task requirements. This required participants to utilize their working memory during the experiments. Thus, our finding of increased theta band power over the parietal area during n-back and target game multi-tasks is in line with other findings from the literature. 

#### 4.1.2. Alpha Band Power

For the primary task, alpha band power increased over parietal and temporal areas but decreased over the frontal area during the more demanding task (suturing). Previous studies have shown that alpha desynchronization (e.g., lower alpha power during the high demanding task) was observed over the task-relevant brain area, whereas synchronization was observed over task-irrelevant brain areas [65,66,67,68]. Our study results were consistent with these studies as we observed that alpha band power decreased over the motor cortex (FP2) (desynchronization) and increased over task-irrelevant areas (CP1, T7, T8) (synchronization) during suturing tasks; these require more demanding motor controls to constantly align the participants’ wrist position with the robot’s end-effector and require fine dexterity and two-handed coordination to stitch a suture. 

For the multi-task requirement, decreased alpha power over the temporal area (T7 and T8) was generally observed for the n-back multi-task compared to the single task condition. From previous studies, alpha band power over parietal areas decreased with increasing n-back levels [69,70]. In this study, desynchronization of alpha power over the temporal area could be caused by auditory stimulus from auditory n-back tasks since the temporal lobe is linked to the auditory cortex. Some studies showed the link between alpha band power over temporal areas and auditory processing [71,72]. For example, Lehtelä et al. found that the presentation of noise induced the suppression of alpha band power over temporal areas. For these reasons, the auditory n-back multi-task could have induced the suppression of the alpha power band in this study. However, studies comparing auditory with other forms of n-back tasks are needed to verify these explanations.

In contrast, increased alpha power over frontal and parietal areas was observed for the target game compared to the single task condition. The target game required working memory to memorize target numbers. Synchronization of alpha was observed over task-irrelevant brain regions, interfering with cognitive processes [66,68]. Some studies have linked this synchronization to an inhibition (interfering processes) of distractors [73,74,75,76]. The studies showed that alpha band power increased over cortical areas responsible for processing distractors. In more detail, the visual target number in the dVRK stereo viewer during target game multi-tasking might be used to visually present cues based on an instructional cueing paradigm [73]. When cues were triggered, participants were instructed to press the pedal while primary tasks (peg transfer/suturing) were considered distractors. In this case, the responsible areas for processing the distractors (primary tasks) were frontal areas (motor cortex). Thus, an increased alpha band power over frontal areas (AF3, FP1, FP2, and FC5) during the target game multi-task could reflect the inhibition process of distractors (primary tasks).

#### 4.1.3. Beta Band Power

In this study, beta band power was not sensitive to differences in the primary tasks. However, beta band power differed between multi-task requirements. When participants were required to do concurrent tasks with the primary task, an increase in beta power was seen in response to the increased workload. Beta band power has been associated with short term memory and visual or auditory stimuli [77,78,79]. Results from this study showed that beta band power during both multi-task requirement conditions increased in comparison to the single task condition. Consistent results were observed in previous studies when additional working memory use was forced on the participants [80,81]. For example, Chen and Huang found increased beta band power with increased working memory load in the visual n-back experiment [80]. However, caution should be taken in using beta band power as a workload indicator because varying beta band powers were observed with an increase in cognitive tasks, and the role of beta band power remains unclear [79]. 

### 4.2. Eye-Tracking

Our results showed that the SF ratio decreased during the more demanding primary task (suturing). SF ratio was used to compare the time spent searching (saccade) to the time spent processing (fixation) [45]. Based on our results, the participants spent relatively more time on processing the task and less time on search activities during suturing tasks compared to peg transfer tasks. This is consistent with the results from previous studies where saccade duration was shorter during the tasks which induced higher cognitive load [82,83], and fixation duration became longer as the task workload demand increased [84,85]. 

However, for the multi-task requirement task effect, a higher SF ratio was observed during the target game multi-task than the single task condition. One potential reason is that the target game forced the participant’s gaze to be dispersed from the primary task to the numerical target numbers on the stereo viewer located at the corner of the field of view. This could have led to a longer saccade duration and shorter fixation duration during the task. 

On the other hand, gaze entropy during target game multi-task was higher than gaze entropy during a single task condition. Gaze entropy is a measure of disordered search processes which do not follow a systematic pattern, and it has been a valid task load index in various areas. Specifically, gaze pattern tends to be more random during more complex workload tasks [86,87]. Other surgical-related studies have also shown that higher gaze entropy is related to higher surgical task load [88,89]. However, a key limitation to using eye-tracking measures in the cognitive workload estimation is that eye behavior patterns depend on the assigned tasks [90,91]. 

### 4.3. Heart Rate Variability (HRV)

Although HRV metrics were not sensitive to the different primary tasks, differences in these metrics were observed for the multi-task requirement effect (n-back task). During the n-back multi-task, a decrease of meanRR and increase of stdRR and RMSSD were observed compared to single task condition. In previous studies for multi-task environments, heart rate, which has a reciprocal relation with the RR interval, increased with task difficulty [92], and HRV decreased during more demanding tasks [85,93]. For example, Veltman et al. observed that adding secondary tasks caused decreased HRV in the flight environment. However, mental workload tasks involving speech tasks should be interpreted with caution because respiratory changes from speech might alter HRV patterns [94,95,96]. In this study, during the n-back multi-task, the participants were required to speak the memorized numbers aloud. Such speech-related respiratory patterns might influence the HRV patterns. Thus, HRV measures used in surgical environments (e.g., RAS) requiring frequent communications between surgical team members need to consider these speech-related respiratory patterns. 

### 4.4. Artificial Neural Network Classification Model

The results of the study show that the classification models had a better performance for the multi-task requirement condition than the primary task condition. One possible explanation for this could be that the addition of the n-back task or target game task as a secondary task in the multi-task condition may have increased the cognitive load and attentional demands on the participants, resulting in more pronounced differences in the physiological signals compared to the primary task alone. Another possibility is that the specific physiological metrics in the classification models were more sensitive to changes in multi-tasking demands rather than primary task factors. In our study, HRV-related metrics were not selected by the Lasso feature selection algorithm as optimal feature sets for the primary task factor, but were included in all levels of the multi-task requirement factors. This suggests that HRV-related metrics may play a more significant role in distinguishing between different levels of multi-tasking demands, potentially contributing to the higher accuracy achieved in the multi-task requirement models.

## 5. Limitations

Several limitations need to be acknowledged in the present study. First, this study application is focused on robotic surgery. However, the study population consisted of university students with no surgical experience. Even though they were trained before the experiments, they do not have the same level of surgical skills as surgeons. However, physiological response patterns to task demands may be consistent across individuals as previous research has shown. Thus, the patterns identified in this study may still provide insight on how physiological responses change with task demands in RAS. Nonetheless, a future study utilizing a surgeon cohort is needed to evaluate the generalizability of these physiological patterns to individuals that perform RAS. Future work is needed to design experimental paradigms with more granular load modulation for evaluating physiological responses during RAS. 

## 6. Conclusions

High cognitive workload experienced in the operating room theater is multifactorial and is better modeled by generating a composite exposure relying on multi-task requirements. This study identified distinguishing physiological responses for two study factors (primary task/multi-task requirement). Multiple responses for EEG band power and channel locations were characterized according to the study factors. Concurrently, we identified gaze pattern distinctions between the two study factors. While the HRV metrics were significant during a multi-task requirement (n-back), they were not sensitive at distinguishing different types of surgical skills (primary task effect). 

Our findings contribute further evidence of the consistencies and differences in physiological behaviors during RAS with existing neuroergonomics work. Understanding how users respond to task difficulty and multitasking workloads can enable the development of robotic surgery interfaces that are responsive to user cognitive states.

## Figures and Tables

**Table 1:** Simulated RAS related surgical tasks.

		Single Task	n-Back Task	Target Game Task
	
Peg transfer		Peg transfer	Peg transfer with n-back	Peg transfer with target game
Suturing		Suturing	Suturing with n-back	Suturing with target game

**Table 2:** Classification model results with identified subset of input variables.

	Classification Model	Identified Features (Lasso)						Accuracy	F-1 Score
		**EEG**				**Eye-tracking**	**HRV**		
		**Theta**	**Alpha**	**Beta**	**Index**				
**Primary task**	**Peg transfer vs. Suturing**	F3, FC6	CP1, FZ	F8	TAR	SF ratio		65.18	66.27
**Multi-task requirement**	**Single task vs. n-back**	AF4, P7, T7	CZ, P3, P7, T7	AF3, CP2, CP5, F3, FC6, FP2, FZ, P4, P8, PO3, T7		Gaze entropy	MeanRR, StdRR, LF/HF	78.61	79.40
	**Single task vs. Target game**	AF4, F4, F7, FC1, FP2, P7, PZ, T7	AF4, CP5, FC6, FP2	AF3, CP2, CZ, FC6,FP1, P7, PO3	TAR	Gaze entropy, SF ratio	MeanRR	75.65	76.44
	**n-back vs. Target game**	F3, FP2, PZ	Cp5, FP2, T7	AF3, FP1, FP2, T7		Gaze entropy	LF/HF	72.39	73.21

## Data Availability

The data that support the findings of this study are available on request from the corresponding author, D.Y. The data are not publicly available due to restrictions containing information that could compromise the privacy of research participants.
